# Acquisition of the Cardinal Principle Coincides with Improvement in Approximate Number System Acuity in Preschoolers

**DOI:** 10.1371/journal.pone.0153072

**Published:** 2016-04-14

**Authors:** Anna Shusterman, Emily Slusser, Justin Halberda, Darko Odic

**Affiliations:** 1 Psychology Department, Wesleyan University, Middletown, Connecticut, United States of America; 2 Department of Child and Adolescent Development, San Jose State University, San Jose, California, United States of America; 3 Department of Psychological and Brain Sciences, Johns Hopkins University, Baltimore, Maryland, United States of America; 4 Department of Psychology, The University of British Columbia, Vancouver, British Columbia, Canada; University of Padova, ITALY

## Abstract

Human mathematical abilities comprise both learned, symbolic representations of number and unlearned, non-symbolic evolutionarily primitive cognitive systems for representing quantities. However, the mechanisms by which our symbolic (verbal) number system becomes integrated with the non-symbolic (non-verbal) representations of approximate magnitude (supported by the Approximate Number System, or ANS) are not well understood. To explore this connection, forty-six children participated in a 6-month longitudinal study assessing verbal number knowledge and non-verbal numerical acuity. Cross-sectional analyses revealed a strong relationship between verbal number knowledge and ANS acuity. Longitudinal analyses suggested that increases in ANS acuity were most strongly related to the acquisition of the cardinal principle, but not to other milestones of verbal number acquisition. These findings suggest that experience with culture and language is intimately linked to changes in the properties of a core cognitive system.

## Background

Number concepts in humans are supported in part by a universal, non-symbolic cognitive system for representing quantities, often referred to as the Approximate Number System (ANS). The ANS is a well-characterized non-symbolic system for representing numerical magnitudes, present from birth in humans [1] and across a wide range of animal species (for review, see [2], [3]). In most cultures, number concepts are also represented by a learned, symbolic system of verbal counting [4], [5]. This paper explores the relationship between culturally-mediated, symbolic mathematical knowledge and the evolutionarily primitive representations of the ANS.

ANS representations are generally characterized on the basis of their internal variability, as manifested in the errors observers make in tasks believed to tap the ANS. For example, ANS representations exhibit scalar variability—with increasing variability as a function of larger set sizes [6]. Accordingly, numerical discrimination tasks yield performance that is a function of the ratio between the two numbers being compared, rather than their absolute difference (i.e., Weber’s law; [7], [8], [9], [10], [11], [12]). In these tasks, the internal variability of the ANS (i.e., ANS acuity) can be estimated through the Weber fraction (*w*)–an estimate of the internal precision of an observer’s ANS representations [9], [13], [10].

A multitude of studies have revealed that individuals’ performance on tests of formal mathematical knowledge is significantly correlated with ANS acuity [14], [15], [16]. For example, numerical acuity in 14-year-olds has been shown to correlate with their performance on standardized math tests in every previous year back to kindergarten [17]. Indeed, ANS acuity is predictive of formal mathematical knowledge by the late preschool years. In one study, acuity measured at 6 months predicted performance on the Test of Early Mathematics Ability, 3^rd^ ed. (TEMA-3), a broad-ranging measure of early mathematical concepts [18]. In the preschool years, ANS acuity correlates with concurrent and future TEMA-3 scores, as well as growth in TEMA-3 scores [14], [19], [20]. Interestingly, an item analysis exploring the relationship between the TEMA-3 and numerical acuity suggests that two items on the TEMA-3, namely understanding of cardinality and arithmetic with physical objects, might carry this effect [21]. These findings clearly suggest a relationship between ANS acuity and mathematical knowledge from early in development, and raise the question of when and how these capacities come to be related.

One important early milestone in symbolic mathematics learning is children’s acquisition of the natural number words (e.g., “one”, “two”, “seven”, etc. [5]). Natural numbers are precise and discrete, and do not show scalar variability, unlike the representations of the ANS. The first step in number word acquisition is learning to recite the count list (i.e. “one”, “two”, “three”*…*). Though most children can do this around age two, children only gradually, over the next two years or so, learn the exact meanings of each of these words. Children first learn the meaning of “one” (but not the other numbers in their count list). Then, after many months, they learn the meaning of “two” and then, after many more months, “three” and then “four”. During this period, children are referred to as subset-knowers, since they have meanings for a subset of their count list [4], [22], [23]. Shortly thereafter, children typically demonstrate an understanding for the next several numbers all at once. This achievement is taken as evidence that they understand the cardinal principle, which stipulates that the position of the final word in a count sequence indicates the cardinal value of the set (i.e., if counting is applied felicitously, the fifth word is “five” and reveals that there are *five* items in the set) [5]. These children are referred to as cardinal-principle (CP) knowers, since they shift from a piecemeal, word-by-word framework to a general understanding of how counting works [23], [24].

In total, it takes two to three years from when children can recite a stable ordered count list from “one” to “ten” to when they can create and identify sets with those quantities [25], [26], [22], [23]. Most researchers agree that children’s initial generalization of the cardinal principle is a central accomplishment of the late preschool years, and that there are important qualitative differences between subset- and CP-knowers [27], [28]. Furthermore, the development of number word knowledge and CP-knower status is related to language development in general, and the timing of this milestone is strongly influenced by general linguistic factors such as vocabulary size [29] and parents’ number talk [30].

LeCorre and Carey [27] hypothesize a further development following the CP induction, which is the development of a strong intuitive sense of the quantities associated with numerals. While children who pass tests of CP knowledge can meaningfully engage the counting procedure in order to establish a set, they do not seem to have well-calibrated intuitions about which set sizes these numerals refer to, typically assayed by their responses on a rapid estimation task (‘Fast Cards’ [27]). Even children who can pass tests of cardinality for “ten” do not produce the number estimate “ten” when briefly presented with ten items on a screen, nor do their mean responses fall along the *x = y* function that defines well-calibrated, proficient estimating. Based on their cross-sectional data, LeCorre and Carey [27] posit that children’s mappings between verbal numbers and ANS representations of magnitudes develop *after* children become CP-knowers, and they differentiate between ‘CP-non-mappers’ who can use meaningful counting but have poor mappings to ANS representations, from ‘CP-mappers’ who have both meaningful counting and ANS mappings under their belts (see also [31], [32], [33]).

Clearly, numerical acuity increases both before and after the years when children acquire verbal counting—on average, the ratio between two numerosities that can be reliably discriminated in behavioral tasks is about 1:2 in six-month-olds [34], 2:3 in nine-month-olds [35], 3:4 in three-year-olds, 4:5 in four-year-olds, and 5:6 in five-year-olds [9], [36]. It is notable, however, that substantial change in ANS acuity occurs contemporaneously with children’s acquisition of meanings for the natural numbers [25], [23], though these two developments have yet to be linked causally.

Several authors have suggested that changes in ANS acuity growth may be related to natural number acquisition (for review, see [2]). One mechanism for such changes may be repeated exposures to number words and formal mathematics throughout development, which result in repeated activation of dedicated neural circuits, leading to gradual increases in the precision, efficiency, or calibration of the ANS [37], [38], [39], [40]. Alternatively, if changes in ANS acuity occur with the initial acquisition of number word meanings, then we might observe a marked increase in ANS acuity corresponding to children’s early acquisition of the number words rather than, or in addition to, any gradual change that comes with prolonged practice. This possibility of a tighter link between children’s initial acquisition of exact number word meanings and improvements in ANS acuity has yet to be demonstrated.

In a previous study, Wagner and Johnson [41] showed a correlation between children’s counting ability and numerical acuity, as measured by accuracy on a dot-discrimination task (e.g., looking at two dot arrays and answering “which has more?”). This initial finding suggests a relationship between cardinal number meanings and ANS acuity, but is not conclusive. First, despite finding a broad correlation between counting and numerical acuity, it is unknown whether individual children experience changes in numerical acuity as they gain each subsequent number word meaning (i.e., as they transition to the next knower level). To answer this question, the current study uses a longitudinal design to search for a tight linkage between counting development and numerical acuity in individual children. Second, Wagner and Johnson [41] and previous studies did not utilize Wynn’s [22], [23] well-defined knower levels framework in relating children’s verbal counting to ANS acuity, so it is difficult to track the precise relationship between verbal number knowledge and acuity. Moreover, because the average age of the children in their study was around 4 years in a high-SES sample, they were likely to be CP-knowers already. Thus their data precludes analysis of how transitions in number word meanings correspond to changes in ANS acuity. This study, therefore, uses a careful titration method to establish knower level for each child in each testing session, with children spanning a range of knower levels. Third, it is unknown which specific transitions in verbal counting, if any, are most related to changes in numerical acuity. This question is best answered with a longitudinal design, which we use here to illuminate which knower level transitions correspond to increased acuity. Similar results were reported by Mussolin, Nys and Leybaert [42], who additionally controlled for IQ and short-term memory, but they used unusual methods to ascertain children’s verbal number knowledge. Thus, these prior studies hint at the relationship between verbal number acquisition and ANS acuity, but leave many questions about how this develops.

We assessed children’s number knowledge and their ANS precision monthly over the course of a six-month longitudinal study and asked whether attaining specific milestones co-occurred with improvements in precision on the ANS task. The milestones we investigated included the learning of individual number word meanings (e.g., *one*, *two*, and *three*, etc.), the induction of the cardinal principle (i.e., understanding that the final word in a count indicates the cardinality of the set), and the successful mapping from ANS representations to number words (e.g., “there are approximately *eight* apples in that picture”).

We assessed verbal number knowledge of exact number meanings using the Give-N task, which was developed by Wynn [22], [23] and has been widely used by many labs studying the early development of number word learning. We assessed the quality of children’s mapping between number words and ANS representations using a rapid estimation task (Fast Cards) adapted from Le Corre and Carey [27]. We assessed ANS acuity with a numerical discrimination task (Panamath [17]) where participants must look at two dot arrays and identify which set has more dots. By using many trials with varying ratios between the two sets, each participant’s threshold for resolving the difference between the array (i.e., their Weber fraction, or *w*) could be computed.

Assuming that at least some children would exhibit transitions in their knower levels during the six-month period, our central question was whether these transitions in number language would be systematically related to the extent and the timing of changes in ANS acuity. To test the relationship between ANS acuity and verbal number knowledge, we conducted a longitudinal assessment of preschoolers’ non-verbal ANS acuity as it changes across transitions in counting ability. The first question was whether parallel growth would be observed in both verbal and non-verbal tasks. A corollary question was whether verbal growth would systematically predict non-verbal growth, or vice-versa, helping to establish a causal direction. The six-month longitudinal design additionally provided an opportunity to replicate three developmental trajectories that have been robustly demonstrated in cross-sectional data. First, we were able to assess individual children’s acquisition of the first few number words, which was first reported in a longitudinal study by Wynn [23] but has not been replicated in a longitudinal study since. Second, these data provide the first longitudinal look at the non-mapper/mapper distinction posited by LeCorre and Carey [27]. Third, this longitudinal design allowed us to investigate the developmental trajectory of ANS acuity in individual preschoolers, which has, thus far, only been posited through cross-sectional comparisons.

## Methods

The following recruitment efforts, testing methods, and general procedures were approved and monitored by Wesleyan University’s Institutional Review Board.

### Participants

Forty-six 3- and 4-year-old children (20 males and 26 females) participated in this study. Prior to enrolling in the study, caregivers signed a statement consenting to their child’s participation in a total of six testing sessions over a 6-month period. Children were also asked to provide oral assent (e.g., “Do you want to play some games?”) before each testing session began. Of the 46 participants, four were unable to complete more than one testing session and were excluded from the following analyses. This left a sample of 42 children (19 males and 23 females) between 36 and 60 months of age (M = 50 months).

Participants were recruited from five urban and suburban daycare and preschool centers in central Connecticut. All children were fluent English speakers. No questions were asked about race or socio-economic background, but participant demographics presumably correspond to the majority Caucasian, middle-class population [43] from which they were drawn.

### Tasks

Each testing session lasted approximately 30 minutes and included three tasks: A measure of ANS acuity (the Who Has More? task [17]), and two measures of verbal number word knowledge (the Give-N task [22], and the Fast Cards task [27]). All of the tasks are described below in the order in which they were completed. [NB: An additional task, the Number Line task, was administered at the end of Session 1 and Session 6 as part of a separate study.] Children received a sticker for their participation at the end of each testing session.

#### ANS Acuity

To determine the precision of the non-verbal number representations supported by the Approximate Number System (i.e., ANS acuity), children completed a numerical discrimination task (similar to that used in Halberda & Feigenson [9]) where two dot arrays were shown briefly and children identified which set had more dots.

An experimenter introduced this task by asking, “Do you want to play a computer game?" Children were presented with a laptop computer (MacBook with 34 cm screen size) displaying two side-by-side rectangles (13 x 16 cm each). The experimenter pointed to the left rectangle and said, “I put my yellow dots in this box,” then pointed to the right rectangle and said, “I put my blue dots in this box.” The experimenter then asked, “Can you please look at the screen and tell me if I have more yellow dots or more blue dots?”

For each trial, a set of yellow dots (solid 2-dimensional circles) appeared in the box on the left side and a set of blue dots appeared on the right. Dot display time was 2500 ms—allowing sufficient experience with the displays while being too short for verbal counting [9]. Children pressed or pointed to a key on the left side of the keyboard marked with a yellow sticker when there were more yellow dots, and a key on the right side of the keyboard with a blue sticker when there were more blue dots. Automated feedback was given in the form of a “ding” for correct responses and a brief “buzz” for incorrect responses, as well as verbal feedback from the experimenter. Children were required to respond on every trial and were encouraged to give their “best guess.” The display went blank when a response key was pressed or when 2500 ms had passed, whichever came first. The next trial did not begin until the child provided a response and indicated she was ready for the next trial.

For the six practice trials, yellow and blue dots were first presented separately and then simultaneously. Throughout the presentation the experimenter would narrate, “Here are my yellow dots, and here are my blue dots. Do I have more yellow or more blue?” Set sizes for practice trials ranged between 4 and 10 dots. The number of dots in the larger set differed from the number of dots in the smaller set by ratios of 1:2 (4 trials) or 2:3 (2 trials). Total surface area co-varied with numerosity for all practice trials. Trial order and the side with the numerically larger set (i.e., the correct answer) varied across trials.

Test trials were identical in structure to the simultaneous portion of the practice trials and began immediately after the series of practice trials. Prompts such as, “Are there more yellow or blue?” were provided by the experimenter when needed. Set sizes were between 4 and 15 dots with ratios ranging from 1:2 to 6:7. For half of the trials, the numerically larger set had more total surface area (area correlated trials) and for the other half of trials the numerically smaller set had more total surface area (area anti-correlated trials). On each trial, the size of the individual dots varied to ensure that individual dot size did not correlate with numerosity (for example, the numerically larger set did not always have smaller dots). Children completed a total of 60 test trials, with ratio and side of the correct response varying across trials.

To calculate individual Weber fractions (*w*), each child’s performance on the ANS acuity task was entered into a psychophysical model which returned a best-fit value for the Weber fraction by Levenberg-Marquardt non-linear least squares fit (see [17]). When we could not fit the psychophysical model to calculate *w*, those data points were excluded, and the analyses were replicated using a measure of general performance (percent correct). We present analyses with both *w* (which are a psychophysical estimate of acuity) and percent correct (which retains all available subjects).

#### Verbal Number Knowledge

The Give-N task [22], [23] was used to evaluate children’s understanding of exact meanings of certain number words. This task began with a brief count elicitation procedure (asking children to count an array of 10–15 small toy fish) to ensure that children could verbally recite the number words “one” through “ten”.

To assess their understanding of individual number words, children were given a set of toy fish and a bowl. On each trial, the experimenter asked the child to put a specified number of fish in the bowl. To ensure that children were satisfied with their responses (e.g., that they did not accidentally grab the wrong number of fish), the experimenter asked the child to confirm (e.g., “Is that five?”). If the child answered “yes,” the next trial began. If the child answered “no” or was unsure, the experimenter asked the child to fix it (e.g., “Can you fix it so that there are five?”). Once fish were added to or removed from the set the child was asked to confirm again.

Children were asked about number words “one” through “eight” according to a titration method. If the child answered correctly (provided the correct amount of fish, *n*), the experimenter asked for *n*+1 fish on the following trial. If the child answered incorrectly, the experimenter asked for *n*-1 fish on the following trial. This continued until the child provided at least 2 out of 3 correct responses for a given number, *n*; fewer than 2 out of 3 correct answers for *n*+1; and avoided giving *n* on trials asking for more than *n* on at least 2 out of 3 trials. This number, *n*, was recorded as the child’s “knower level” and served as a measure of the child’s understanding of the verbal cardinal numbers [23]. Children demonstrating an understanding of only a subset of the number words tested were sorted into the *one-*, *two-*, *three*-, or *four*-knower levels. The *Cardinal Principle*-knower (CP-knower) level comprised children who succeeded on all set sizes as these children were able to use counting in order to determine the number of items in a set, thereby demonstrating an understanding of the cardinal principle.

A version of the Fast Cards task [27] was used to evaluate children’s mapping of number words to approximate numerosities. Using the same laptop computer as the ANS acuity task, children viewed a series of PowerPoint slides displaying sets of objects (trains, fish, monkeys, hats, or snowflakes).

Children first completed fifteen practice trials showing one to fifteen fish, presented in numerical order. The experimenter asked, “What is this?” and responded to the child by saying, “This is a number game, so for this picture we would say *one*.” The remaining practice trials were modeled by the experimenter unless the child caught on and could continue independently. Two of the test blocks held total surface area constant, allowing individual item size to shrink as numerosity increased, and the other two blocks held item size constant, allowing total surface area (i.e., total pixels taken up by the items) to increase with increasing numerosity.

Test trials began directly after the practice trials. Children completed four blocks of seven test trials, presented in a fixed order. Each block included sets of 1, 2, 3, 4, 6, 10, and 14 objects, with a different fixed random order for each block. Display times were limited (1000 ms each trial) to prevent counting. If children began counting aloud, experimenters said, “This is not a counting game, just say what number word goes with the picture.” Experimenters recorded the number word provided by the child for each trial.

Following Le Corre and Carey [27], CP-Knowers (classified by the Give-N task) were further sorted into two groups based on two criteria: CP-*Mappers* (1) provided higher number words for larger sets, thereby producing a positive slope greater than 0.3, and (2) provided significantly higher mean estimates for 14-item trials than 6-item trials. Children who did not meet these criteria were classified as CP-*Non-Mappers*. No subset-knower (i.e., one-, two-, three-, or four-knower) showed a profile consistently meeting the Mapper criteria, so these categories were only applied to CP-Knowers. See S1 DataSet in Supporting Information for the complete dataset.

## Results

### Overview

Our primary goals were to (1) document longitudinal changes in verbal number word knowledge, (2) document longitudinal changes in ANS acuity, and (3) determine if transitions in number word knowledge systematically varied with changes in ANS acuity. We first present a series of cross-sectional analyses of verbal and non-verbal measures related to these goals. We then present longitudinal analyses of each child’s verbal number knowledge, ANS acuity, and the relationship between them.

### Cross-Sectional Analyses

Cross-sectional analyses were used to document performance on each task and to assess associations between the different measures. To this end, we created two samples where each child contributed one data point from the first half of the study (Sessions 1–3) and one from the second half (Sessions 4–6). When available, data from the first half of the study were sampled from Session 2 and data from the second half of the study were sampled from Session 5, with the midpoint session taken to be representative of each half of the larger study. If Session 2 or 5 data were not available, an adjacent session was used instead (e.g. Session 4 instead of Session 5).

Session 2 (S2) analyses include all 42 children (19 males, 23 females) ranging from 38 to 60 months of age (M = 48 months). Session 5 (S5) analyses include 39 children (18 males and 21 females) ranging from 39 to 59 months of age (M = 51 months; the remaining three children did not complete any of the last three sessions and were thus excluded from S5 analyses). Each child contributed their knower level (one-knower, two-knower, three-knower, four-knower, CP-Non-Mapper, or CP-Mapper, assessed through the Give-N and Fast Cards tasks) as well as two measures of ANS acuity (assessed through the Who Has More task): general performance (percent correct) and *w*. Data from five children (n = 4 in S2, n = 1 in S5) did not fit the psychophysical model and were excluded from analyses of *w*. All other reports, including the percent correct analyses, incorporate all children in this sample.

#### Verbal Number Knowledge

All children could accurately count up to ten with no more than one error. Using their performance on the Give-N and Fast Cards tasks, children were sorted into the following knower levels: one-knower, two-knower, three-knower, four-knower, CP-Mapper, or CP-Non-Mapper (see Tables 1 and 2 for the number and ages of children classified in each level). Replicating a general trend noted in nearly all studies evaluating children’s knower levels (see [44] for a meta-analysis including over 600 2- to 4-year-old children) we observed that children’s knower level significantly increases with age, Spearman rank correlation, r_s_(41) = .55, p < .01 at S2 and r_s_(38) = .51, p < .01 at S5. A one-way ANOVA, with sex as the between subjects variable and knower level as the dependent variable, reveals an interesting trend with females achieving higher knower levels than males, F(1, 41) = 3.32, p = .08 at S2 and F(1, 38) = 4.17, p = .05 at S5 (when controlling for age, F(1, 41) = 3.72, p = .06 at S2 and F(1, 38) = 3.12, p = .09 at S5). Subsequent analyses of verbal number knowledge therefore control for both age and sex.

**Table 1:** Distribution of Children across Knower Levels at Session 2. Measures of numerical acuity, *w* and percent correct, assessed through the Who Has More task.

	Session 2 (S2)						
Knower Level	N	Age Mean *(mo)*	Age Range *(mo)*	w-Score Mean	w-Score SD	% Correct Mean	% Correct SD
One Knowers	4	40	38–43	.73	.43	57.5	13.4
Two Knowers	7	47	38–54	.75	.55	61.9	14.5
Three Knowers	4	47	40–53	.62	.39	70.0	6.4
Four Knowers	4	42	39–44	1.56	1.09	60.8	8.7
CP Non Mappers	11	50	45–54	.41	.23	73.8	9.4
CP Mappers	12	52	45–54	.37	.29	76.3	8.4
**Totals**	**42**	**48**	**38–60**	**.60**	**.56**	**69.4**	**11.9**

**Table 2:** Distribution of Children across Knower Levels at Session 5. Measures of numerical acuity, *w* and percent correct, assessed through the Who Has More task.

	Session 5 (S5)						
Knower Level	N	Age Mean *(mo)*	Age Range *(mo)*	w-Score Mean	w-Score SD	% Correct Mean	% Correct SD
One Knowers	1	41	*n/a*	1.42	*n/a*	53.3	*n/a*
Two Knowers	5	48	39–54	1.04	.50	64.0	8.3
Three Knowers	3	45	40–48	.92	.41	58.9	9.2
Four Knowers	2	45	43–47	.58	.42	75.0	7.1
CP Non Mappers	21	52	42–59	.43	.27	74.1	9.9
CP Mappers	7	54	49–56	.27	.15	81.4	10.0
**Totals**	**39**	**50**	**39–59**	**.54**	**.40**	**72.5**	**11.4**

#### ANS Acuity

**Fig 1:**
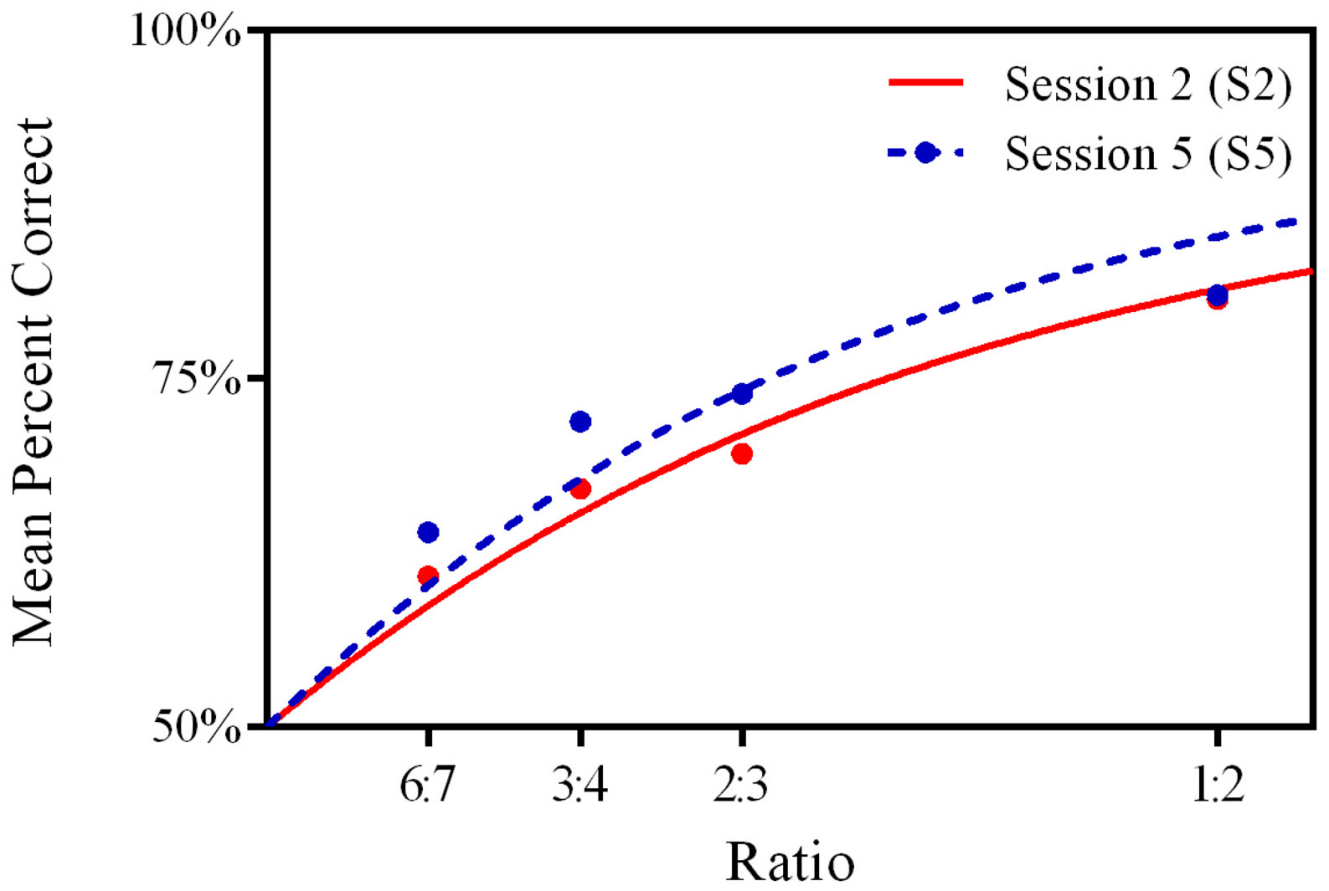
Children’s Performance on the Who Has More Task. Lines show psychophysical model fit.

Performance varied as a function of the ratio of the two set sizes being compared, with performance (percent correct) dropping as ratios approached 1 in both S2 and S5, replicating the signature profile of the ANS (see Fig 1): A one-way ANOVA with percent correct as the dependent variable and repeated measures on ratio (1.17, 1.33, 1.50, 2.00) at each time point was significant, F(3, 41) = 16.06 at S2, p <. 01, F(3, 38) = 12.54, p < .01 at S5. Age and ANS acuity (*w*) were significantly correlated at S2 (r_s_(38) = -.36, p = .03) and marginally associated at S5 (r_s_(38) = -.28, p = .08), with older children exhibiting better ANS acuity. Similarly, age was significantly correlated with percent correct on the ANS acuity task at S2, r_s_(42) = .43, p < .01, but not at S5, r_s_(39) = .26, p = .11. Generally speaking, this trend is consistent with previous findings showing that ANS acuity improves with age [9], [45], [36], [38].

We next look at effects of sex and visual array type (area correlated, area anti-correlated). A one-way ANCOVA with sex as the between subjects variable, *w* as the dependent variable, and age as a covariate shows that ANS acuity at S2 was slightly better for females (n = 21, M = 0.48) than males (n = 17, M = 0.75) at S2 (F(2, 37) = 2.62, p = .09), and significantly better for females (n = 20, M = 0.43) than males (n = 18, M = 0.65) at S5 (F(2,37) = 4.27, p = .02). A paired samples t-test with percent correct as the dependent variable shows that mean performance for area correlated trials did not significantly differ from performance for area anti-correlated trials at S2 or S5, t(41) = 1.86, p = .07 and t(38) = 0.62, p = .54, respectively. Because significant effects of age and sex were found, these variables were controlled for in subsequent analyses. Given that children did not consistently respond differently to area correlated and anti-correlated trials, this variable was not factored into subsequent analyses.

#### Relationship between ANS Acuity and Verbal Number Knowledge

**Fig 2:**
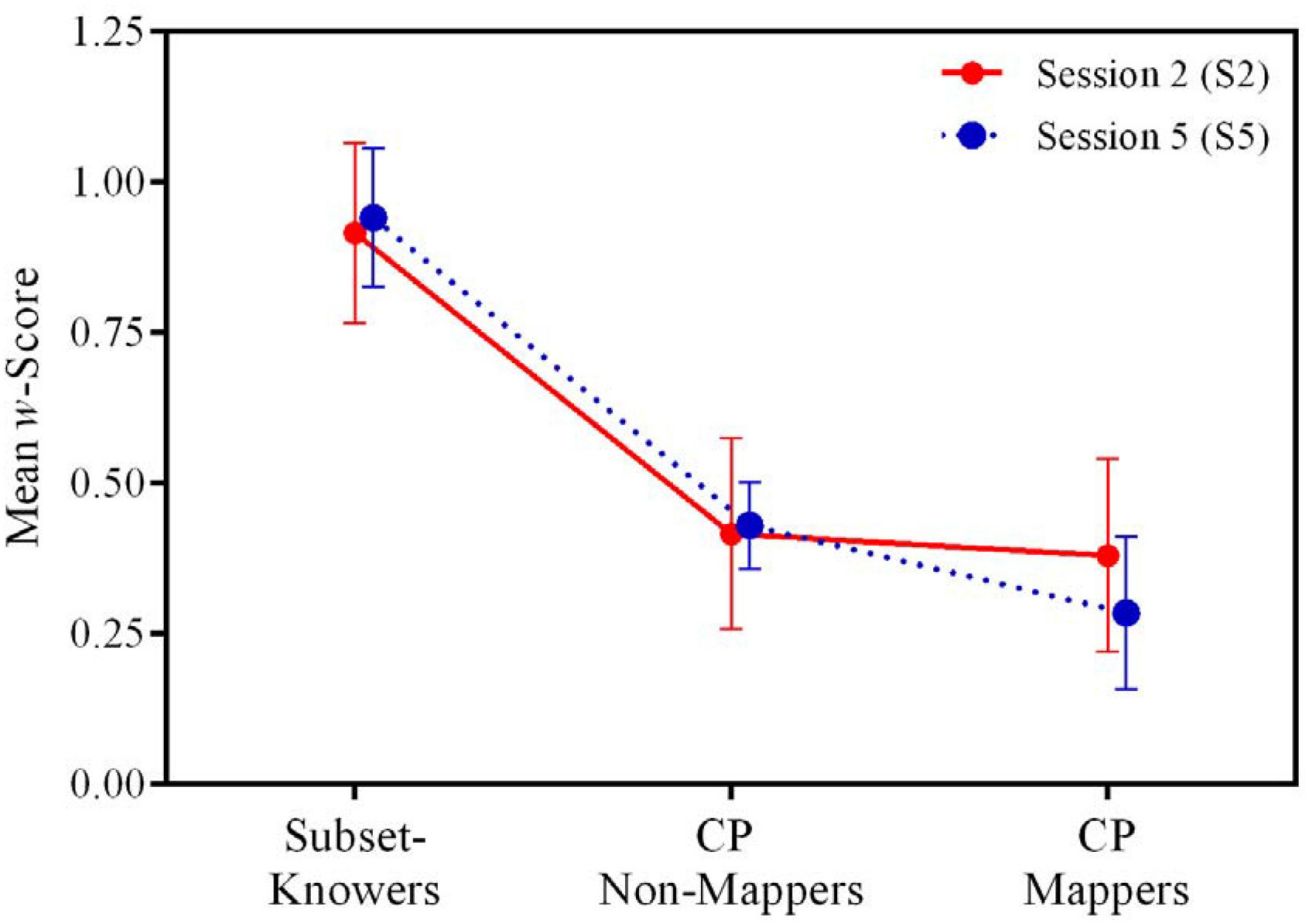
Estimated Marginal Means of ANS Acuity as a Function of Knower Level. Error bars represent +/- 1 SE.

A one-way ANCOVA, with knower level (one-knower, two-knower, three-knower, four-knower, CP-Mapper, CP-Non-Mapper) as the between subjects variable, *w* as the dependent variable, and age and sex as covariates, revealed a significant effect of knower level on *w* both at S2, F(5, 37) = 4.00, p < .01, and at S5, F(5, 37) = 4.43, p < .01. Tukey post hoc tests indicated significant differences at both sessions between subset-knowers and both levels of CP-knowers, p < .05 for all four comparisons, but not between CP-Mappers and CP-Non-Mappers. The same effect was found when evaluating percent correct as a measure of ANS performance at S2, F(5, 41) = 2.72, p = .02, and at S5, F(5, 38) = 2.79, p = .02. Thus, after controlling for sex and age, children with stronger counting knowledge also showed better numerical acuity (Fig 2, see also Tables 1 and 2). This is, to our knowledge, the first demonstration of a relationship between knower level and ANS acuity when controlling for age and sex.

In sum, the cross-sectional analyses suggested that both knower level and numerical acuity increased with age, and that these measures were correlated with one another, even when accounting for the covariates of age and sex.

### Longitudinal Analyses

Given the cross-sectional results showing that knower level and ANS acuity were correlated with age *across* children, we next used the longitudinal design to assess whether this pattern holds *within* children. We then test the hypothesis that discrete changes in knower level are associated with increases in ANS acuity.

#### Verbal Number Knowledge

Of the 42 children who completed two or more testing sessions, the verbal number knowledge of 30 children improved by at least one level over the 6-month testing period. These improvements, or knower level “jumps”, were recorded whenever children advanced from one subset level to another (n = 11), from a subset level to the CP level (n = 7), or from the CP-Non-Mapper to CP-Mapper level (n = 12), from their previous testing session. Of the 12 children who did not jump to a higher knower level, six performed at ceiling (i.e. they were classified as a CP-Mapper) at the first test session. Only six children did not show increases in verbal number knowledge over the course of the study (four subset-knowers and two CP-Non-Mappers).

To see whether changes in verbal number knowledge were robust, we also looked for “drops” in knower levels across sessions. Using the knower level categories from the Give-N task, only one drop was recorded (one child dropped from a three-knower to a two-knower). Thus, as in Karen Wynn’s original longitudinal study [23] of growth in verbal number knowledge, shifts in children’s number word meanings were generally stable. In contrast, a total of 16 drops from CP-Mapper to CP-Non-Mapper were recorded over the course of the study, suggesting a qualitative difference between this conceptual shift and knower level jumps. This result bears highlighting as it is the first longitudinal assessment of the stability of the putative CP-Non-Mapper to CP-Mapper shift [27].

#### ANS Acuity

A one-way ANCOVA, with *w* as the dependent variable, repeated measures on session (S2 and S5), and baseline age and sex as covariates, showed that *w* did not significantly improve within subjects over the 6-month period, F(1, 32) = 0.31, p = .58 (5 children did not contribute a *w* score at either S2 or S5 and were excluded from this analysis). Similarly, no effect was detected when evaluating percent correct as a measure of ANS performance, F(1, 38) = 0.33, p = .57.

This longitudinal study provided an opportunity to assess the test-retest reliability of the ANS acuity task in this age range. The Pearson correlation between *w* at S2 and S5, based on this task, was r(38) = .46, p < .001.

#### Relationship between ANS Acuity and Verbal Number Knowledge

Longitudinal analyses of verbal number learning and ANS acuity indicated that children’s performance clearly changed on both measures, but not linearly; for example, knower levels exhibited jumps and plateaus rather than continuous change. [NB: Although change in performance on the ANS task over time was not detected in the S2–S5 analysis reported above, such change was confirmed using a hierarchical linear model (HLM), which indicated a significant linear increase in percent correct responses across the six sessions (β = 0.81, p = .01). HLM analyses did not reveal a corresponding linear change in *w*.] Therefore, we focused our analyses on discrete, non-linear transitions in both measures so as to understand the nature of the correlation between ANS acuity and verbal number knowledge.

**Fig 3:**
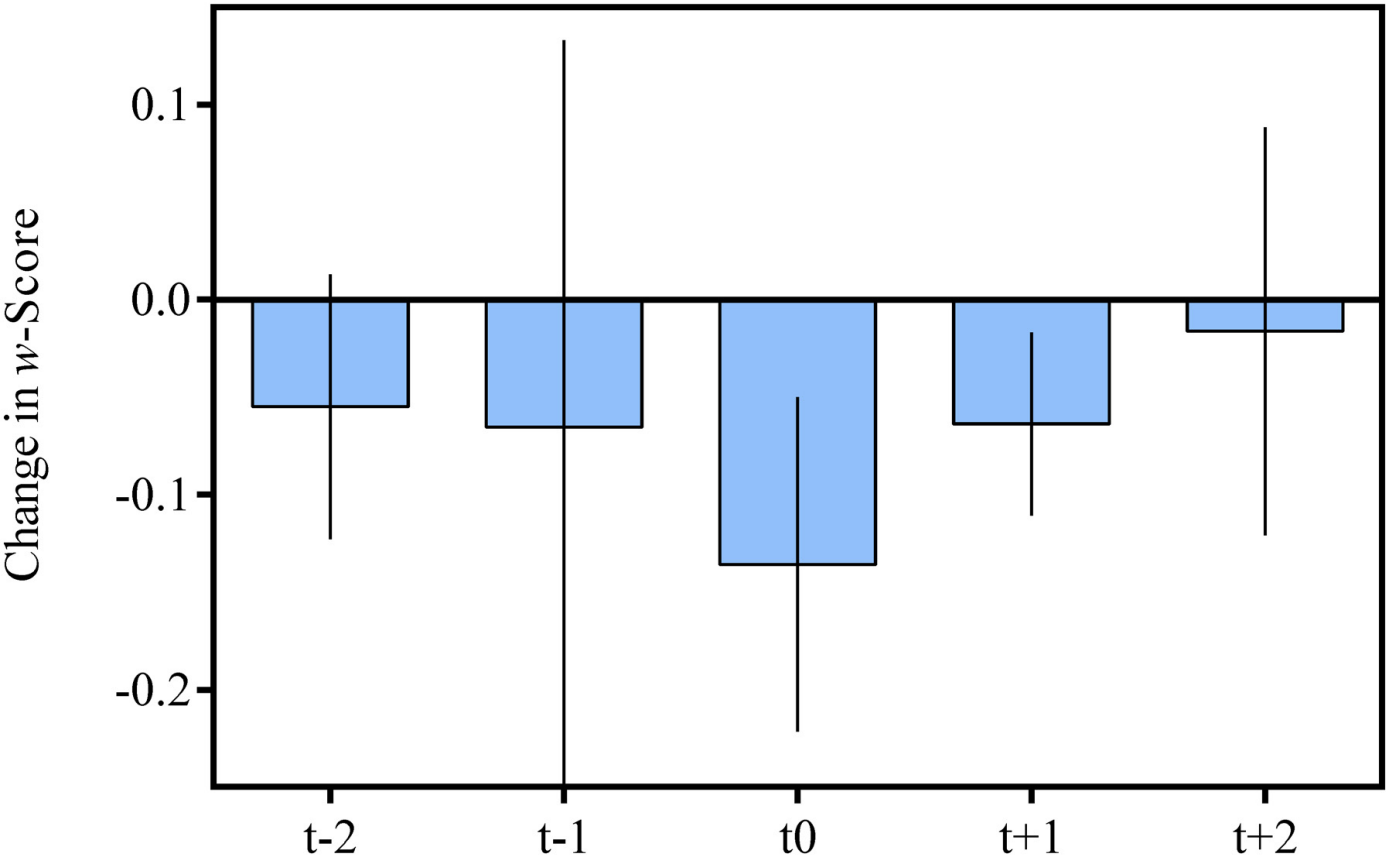
Mean Change in w-Scores Across Sessions. [t0] represents the session at which a jump between knower levels occurred. [t-2] and [t-1] represent sessions preceding the jump and [t+1] and [t+2] represent subsequent sessions. Error bars represent SEM.

What is the timing of developmental transitions in verbal number and ANS acuity? First, we isolated the test sessions corresponding to improvements in verbal number (i.e., the knower level jumps described above) and classified children in groups according to these jumps. Three children made a jump across knower levels that occurred across non-consecutive testing sessions spanning more than one month (e.g., tested in session 2, absent for testing in session 3, jump noted in session 4). Because we could not isolate the point at which the advance was made, these children were excluded from the reported analyses, leaving 27 jumps for analysis. The final groups were: Subset-to-Subset jumps (n = 9), Subset-to-CP jumps (n = 6), and CP-Non-Mapper-to-CP-Mapper jumps (n = 12). The sessions where a jump was first observed were coded as [t0] and adjacent test sessions were coded as [t-1] or [t+1]. For example, if a child’s transition from a Subset- to-CP-knower was revealed at the fourth testing session, this session would be coded as [t0]. Session 3 and Session 5 would be coded as [t-1] and [t+1], respectively. We then computed the change in ANS acuity between each session. As shown in Fig 3, the largest changes in mean ANS acuity (*w*) were coincident with these knower level jumps (t[0] Δ_*w*_ = -.14), while changes in ANS acuity for sessions immediately before and after this session were, on average, only half as large (t[-1] Δ_*w*_ = -.07 and [t+1] Δ_*w*_ = -.06). While these differences were not statistically significant (p > .05) due to the small sample and large variance, the data hint at a possible relationship between jumps in verbal number word knowledge and ANS acuity. This observation prompted a closer look at the t[0] sessions for discernible patterns, namely differences between subsets of children exhibiting jumps in verbal number.

Are certain verbal number milestones particularly related to improvements in ANS acuity? We next asked if children exhibit changes in numerical acuity during each development in number word knowledge, or, alternatively, if such changes are more pronounced during particular improvements in number word knowledge (e.g., changes within subset-knower levels versus change from subset- to CP-knower). To this end, a one-way Repeated Measures ANOVA tested whether numerical acuity differed as a function of the jump in verbal number. The independent variable was Jump Category (Subset-to-Subset, Subset-to-CP, and CP-Non-Mapper-to-CP-Mapper) as the between-subjects measure. The dependent variable was a repeated measure: *w* on the ANS task at session [t-1], the session just prior to the jump, and at [t0], the session at which the jump was observed. The Session x Jump Category interaction effect was significant, F(2,16) = 4.66, p = .03 (n = 19), indicating that different advances in children’s verbal number knowledge were differentially related to changes in ANS acuity. A post-hoc analysis revealed that the Subset-to-CP group showed the biggest change (Δ_*w*_ = -0.455), relative to the Subset-to-Subset (Δ_*w*_ = 0.001) and CP-Non-Mapper to CP-Mapper (Δ_*w*_ = -0.069); however, only the difference between the Subset-to-CP group and the Non-Mapper-to-Mapper group was statistically significant (p = .02), while the difference between Subset-to-CP and Subset-to-Subset was not (p = .19).

Because the strict criteria necessary for calculating *w* resulted in missing data points (n = 10) for critical sessions, we repeated the ANOVA with all participants using percent correct as the dependent measure. Using percent correct, the interaction between Session x Jump Category was significant, F(2,26) = 13.55, p < .01, with the same pattern in the post-hoc analysis as was seen in the *w* analysis.

The above analysis focused on t[0], the exact time point of change, given the initial observation that the biggest changes in *w* corresponded to t[0]. The observation that the Subset-to-CP group showed the largest changes in *w*, and percent correct, motivated us to verify this finding with additional analyses. A second analysis looked at children’s jumps between S2 and S5 (the time slices used in the cross-sectional analyses above). A one-way ANCOVA with Jump Category as the between-subjects factor, and a repeated measure, *w* at Session (S2, S5) as the dependent measure, with age and sex as covariates, revealed a significant interaction of Session and Jump Category, F(1,29) = 3.89, p = .05. A post-hoc analysis using Tukey’s HSD criterion indicated that those children who jumped from a subset-to-CP-knower level exhibited the largest changes in ANS acuity (Subset-to-CP group, n = 7, M = -.41; compared to the Subset-to-Subset group, n = 7, M = -.04, p < .05; compared to the CP-Non-Mapper-to-CP-Mapper group, n = 12, M = -.18, p < .05; and compared to the No Change group, n = 9, M = .10, p > .05). [NB: One additional child in the Subset-to-CP group contributed data to this S2-to-S5 analysis, but did not have data from adjacent sessions for the t[0] analysis described earlier. This is why there are 6 children are available for the t[0] analysis but 7 for the S2-to-S5 analysis.]

**Fig 4:**
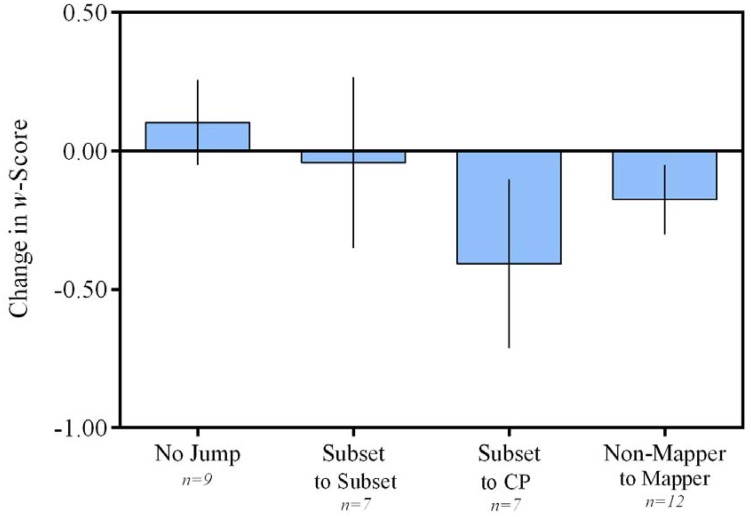
Mean Change in w-Score from Session 2 to Session 5. ANS acuity undergoes the greatest change for children jumping from a subset- to CP-knower level. Error bars represent SEM.

A parallel analysis using S2 and S5 percent correct scores as the repeated measure similarly showed a significant interaction of Session and Jump Category, F(1,33) = 10.69. p < .01 with post-hoc Tukey’s HSD again indicating that the subset-to-CP-knower group exhibited the largest gain in percent correct (Fig 4; Subset-to-CP group, n = 7, M = 6.11%; compared to Subset-to-Subset group, n = 10, M = 3.54%, p < .05; compared to the CP-Non-Mapper-to-CP-Mapper group, n = 12, M = 4.44%, p < .05; and compared to the No Change group, n = 10, M = 2.17%, p > .05).

Thus, different jumps in verbal number knowledge were related to distinct effects in increases in children’s ANS acuity. In all analyses, jumping from subset to CP-knower level corresponded to the largest increases in performance on the ANS acuity task.

A final analysis took a closer look at the six children who made a subset-to-CP jump during the study and changes in their ANS acuity. For three children, the largest improvement in ANS acuity during the 6-month study happened at [t0] (using *w*), coincident with their shift in knower levels (using percent correct rather than *w*, the largest changes for these children were at [t0], [t+1], and [t-2]). Two children could not be fitted with a *w* at [t-1] and their largest increases were observed at [t+1] (using percent correct instead of *w*, the biggest changes for these children were at [t0] and [t+2]). One child’s largest increase was observed at [t+2], on both *w* and percent correct measures. Because this subgroup was small and the individual data were noisy, it is not possible to draw strong conclusions about the temporal order of shifts of verbal relative to non-verbal number development. Nevertheless, it is striking that all six children who acquired the cardinal principle also consistently showed a robust change in numerical acuity once they demonstrated mastery of the cardinal principle or in the few months following this conceptual leap.

## Discussion

While many animals share with humans a primitive Approximate Number System (ANS), only humans naturally acquire an understanding of number symbols and large exact cardinalities. We set out to test the possibility that the protracted process of learning meanings for numerals is related to developmental change in ANS acuity. Our findings support this hypothesis in two ways. First, the cross-sectional analyses reveal that children with more advanced verbal number knowledge also had better numerical acuity, even after controlling for age. Second, and even more surprisingly, the longitudinal analyses reveal that individual children’s growth in verbal number knowledge corresponded to jumps in ANS acuity. In both analyses, understanding larger exact cardinality terms (e.g., “five”)–the hallmark achievement of early number word learning—was the milestone most strongly related to increases in ANS acuity.

The cross-sectional results extend previous findings on the relationship between symbolic math and ANS acuity. Libertus et al. [14] demonstrated an association between a broad test of early mathematical knowledge, the TEMA-3, and ANS acuity in preschoolers. While this and related studies [46], [19], [47], [18] have established a link between preschool mathematical knowledge and ANS acuity, it has not been clear what aspects of symbolic mathematical knowledge underlie this relationship. The current study highlights children’s understanding of the cardinal principle as a critical conceptual leap associated with robust improvements in the precision of nonverbal number representations. While ANS acuity may have improved for many children over the duration of the study, the onset of the cardinal principle was associated with the greatest improvements in ANS acuity. In fact, jumps from one subset-knower level to another did not systematically co-occur with jumps in ANS acuity, suggesting that the transition to understanding the cardinal principle may be uniquely related to improved ANS acuity.

These findings also refine theoretical approaches to the relationship between number thought and number language. First, these results indicate a strong relationship between the acquisition of counting words and nonverbal number representations during typical development. Previous studies looking for such associations have yielded mixed results. For example, Rousselle and Noël [48] used a large-number dot-comparison task, similar to the current study, but found no correlation between performance on a Point-to-X verbal number task and a non-verbal estimation task. Most of the children they tested, however, already responded confidently for numbers “four” and higher, suggesting that they were likely CP-knowers. Thus, variation in children’s knowledge of numbers higher than “four” (i.e., “five” to “twelve”) might *not* co-vary with ANS acuity. Similarly, Huntley-Fenner and Cannon [49], in an early demonstration of the ANS in preschoolers, also found no correlation with verbal counting using assessments of how high children could count. This is likely because such counting tasks are believed to measure rote memorization rather than an understanding of cardinality [25]; in addition, nearly all of the children were the same age or older than typical CP-knowers, suggesting again that variation in verbal counting beyond the acquisition of cardinality is not strongly correlated to numerical acuity. It is also important to note the potential increased power provided by the longitudinal design used here, which allowed us to pinpoint a tight temporal relationship between individual children’s attainment of understanding the cardinal principle and improvements in ANS acuity.

Studies that *have* reported a positive relationship between the acquisition of verbal counting and non-verbal performance have used stimuli with small set sizes [50], [51], [52]. However, small set sizes may elicit responses based on a different cognitive system for enriched parallel individuation rather than responses based on ANS representations [53], [27], [54]. Mix [52], as well as Brannon and Van de Walle [50], found the greatest differences in non-verbal number task performance between children who could not count at all and those who had broken into the counting system through learning a meaning for at least “one”. Thus, initially learning labels for the small sets, around age two, might foster more allocation of attention to numerosity as opposed to other perceptually salient cues, resulting in a marked improvement on non-verbal tasks. The current data suggest that, approximately two years later, induction of the cardinal principle represents a separate developmental shift, with a corresponding improvement in ANS acuity.

The results presented here additionally provide a strong replication of Wynn’s original longitudinal study [23] in which she reported that knower levels in number acquisition are robust and stable. While many researchers have found support for the claim that children pass through discrete knower levels in number acquisition using cross-sectional data [26], the current results solidify this claim using repeated measures over a series of six testing sessions with a relatively large sample size for such studies.

Surprisingly, the distinction between CP-Mappers and CP-Non-mappers, proposed by LeCorre and Carey [27] on the basis of cross-sectional estimation data, did not emerge robustly in these longitudinal data and were not associated with ANS acuity. Using Le Corre and Carey’s [27] criteria, children were straightforwardly classified into these categories in any given session, but fluctuated between them across sessions. One possibility is that the Non-Mapper/Mapper distinction tapped performance variables, such as attention during a particular session, rather than true conceptual stages. Another possibility is that different criteria, other than LeCorre and Carey’s [27] original standards that we followed here, might yield a more consistent transition from Non-Mapper to Mapper; however, we have been unable to identify any such criterion in this data set despite extensive efforts [55].

The current data raise a number of further questions. One question is whether changes in children’s responses to the ANS acuity task reflect changes in the underlying ANS representations or other aspects of their attention or response biases. For example, children who understand cardinality might attend more to discrete numerosity instead of task-irrelevant cues (such as total spatial extent). Note that we observed no consistent difference in performance between trials where surface area was congruent with numerosity and trials where it was not, suggesting that surface area was not a distracting cue for children in this study. Nevertheless, other task-irrelevant factors might be more distracting to less numerically-competent children. Additionally, one might hypothesize that some children have less understanding of what the word “more” means in the ANS acuity task instructions (“Which side has *more* dots?”), as argued in a recent paper by Negen and Sarnecka [56]; but see Odic, Pietroski, Hunter, Lidz, & Halberda [57] for evidence that children know what “more” means, in the comparative sense used here, by age 3. While it may be the case that less numerate children have difficulty interpreting or following the task instructions, confusion about “more” cannot explain our results. Children had multiple sources of information about the task, with feedback on every trial reinforcing the selection of the more numerous side. Children who did not understand the task at all would perform at chance, and would not show the ratio effect necessary for generating a *w*. We observed chance performance in some children (noted in the Results as a subset of the children for whom the *w*-model did not settle on a value), yet many subset-knowers performed above chance, fit the psychophysical model, and contributed to the *w* analyses. Thus, a potential failure to understand the semantics of “more” cannot explain the observed relationship between performance on the acuity task and verbal number knowledge.

A further question is whether the dot-comparison task is an ideal measure of ANS acuity in preschoolers. Those children who did respond at chance at easily discriminable ratios may not know what is being asked of them, despite getting feedback on every trial. Additionally, the variation in individual children’s performance on the task across testing sessions (e.g., r = .46 from S2 to S5) indicates a need to improve the stability of this measure of ANS acuity in young children. New methods of assessing ANS acuity in preschoolers, especially measures that do not require an explicit behavioral response (e.g., ERP, NIRS, or eye-tracking measures) could be useful for drawing conclusions about the quality of ANS representations. For interpreting the relationship between number word knowledge and ANS acuity reported here, it is noteworthy that any additional noise in our ANS acuity measure would limit our ability to detect these effects and, therefore, makes it all the more striking that the acquisition of the cardinal principle significantly correlated with improvements in ANS acuity.

The most important question raised by these results is why we might observe a strong relationship between ANS acuity and verbal number. Here we lay out, briefly, four distinct ways that such relationships might form, noting that they are not necessarily mutually exclusive.

First, according to some researchers, the ANS serves as a source of number word meanings [58], [59], [6], [60]. On this hypothesis, maturational change in ANS acuity supports the acquisition of number language. That is to say, if the quality of ANS representations improve such that quantities are more discriminable from each other (e.g., sets containing *five* and *six* items become easier to distinguish from one another), it becomes easier for that child to map number words like “five” and “six” to their appropriate ANS representations. The underlying mechanism in this hypothesis is an associative mapping between number words and probabilistic ANS representations of cardinal quantities (i.e., “blurs on the mental number line”). In support of this position, proponents of this view cite evidence that children map ANS representations to their initial number word meanings, at least in a rudimentary way, one to two years before they acquire the cardinal principle and understand counting [31], [60], [41], [61], [27].

However, there are also sound arguments against this view. For example, while Huang et al. [60] showed that three-knowers who are trained on “four” perform better in test trials where *four* is contrasted with a large set (10) than with a small set (5), a magnitude effect that is loosely consistent with the ratio-signature of the ANS, the effect was not replicated in a follow-up study by Carey et al. [61]. Furthermore, recent studies by Odic et al. [33] and Gunderson et al. [31] demonstrate that, while some children do form some mappings between numerals and non-verbal ANS representations prior to becoming CP-knowers, other children form such mappings only after becoming CP-knowers (see also [27]). Therefore, mappings to ANS quantities are unlikely to be a *necessary* foundation for the meanings of number words. Nor can mappings between numerals and the ANS can be *sufficient* for providing meanings for number words, because number words denote distinctions, like the one between *seventy-eight* and *seventy-nine*, that are, in practice, well beyond the resolution of the ANS. Finally, a series of training studies by Carey et al. [61] showed that young CP-knowers fail to learn to associate the word “ten” with a set of 10 objects, when given structured training; if children learned number words like “ten” by mapping them onto ANS representations of quantities, then children who understand cardinality and can count to “ten” should form such mappings fairly easily, yet they do not (see also [62], [63] for a different argument that structure mappings, not associative mappings, characterize the mapping between the ANS and the count list). In short, despite the pervasiveness of this idea in the literature, the weight of the evidence does not support the claim that all of children’s initial verbal number word meanings are mapped to regions of a ‘mental number line’ instantiated in the ANS.

On the surface, the data here might be seen as consistent with the view that ANS representations underlie number word meanings. As would be expected on this view, an increase in ANS acuity co-occurs with the acquisition of cardinality. However, Hypothesis 1 would also predict a strong relationship between ANS acuity and accurate mappings between sets and number words, as measured by the Fast Cards task. This relationship between mapping and acuity is not observed, further suggesting that Hypothesis 1 is an unlikely explanation for the current data.

The second view (Hypothesis 2) entails a more general relationship between the ANS and number words, and does not posit associative mappings between specific numerals and specific ANS representations. For example, ANS acuity in infancy predicts the acquisition of symbolic mathematical reasoning [18]. Relatedly, practicing with tasks that engage the ANS yields positive, though modest, effects on performance on speeded symbolic math tests in children and adults [64], [65]. While neither training study shows evidence that such practice increases ANS acuity, both studies suggest that engaging the ANS can boost symbolic mathematical reasoning. Thus, changes in the ANS may have consequences for numeral learning: for example, enhancements in ANS acuity and efficiency could make it easier for children to think about multiple quantities simultaneously, which could in turn facilitate children’s understanding of the relational structure of the count list. To date, however, there is not sufficient evidence to evaluate the causal consequences of ANS training or improvement on number word learning in young children.

On the third and fourth hypotheses, ANS representations for specific quantities could themselves change as a result of children’s learning about number words. Previous studies have demonstrated that the ANS is malleable with training or feedback [37], [66]; natural number acquisition could serve as one relevant type of experience that influences the ANS. Empirical studies comparing the performance of numerate and innumerate populations on purely non-symbolic tasks show that participation in a numerate culture changes the quality of ANS representations [67], [38] as well as memory for discrete quantities [68], [69], [70], supporting the claim that exposure to a numerate culture with a count list may causally influence changes in non-verbal representations.

Hypothesis 3 is that such changes would be specific to certain numbers: Number acquisition could lead to sharpening ANS representations of the specific quantities for which the number words were learned. For example, experience with counting or conceptual leaps in the understanding of number words could induce a narrowing of tuning curves around ANS representations (for computational accounts, see [58], [71]). Contrary to this suggestion, while the jump to CP-Knower corresponded to the greater improvements in ANS acuity in our sample, the ANS acuity task itself involved many trials with numbers of items beyond the children’s counting ability. This is suggestive evidence against Hypothesis 3, though more focused testing on specific numbers is necessary.

Hypothesis 4 is that learning language for numbers could increase ANS acuity in a broad sense, either by changing the quality of the system or by facilitating attention or efficiency for processing quantities in the ANS. On this view, acquiring number language would not be beneficial solely as a ‘tool for thought’ to encode the cardinal value of a set [69] or as a way to fine-tune ANS representations for specific quantities [71]. Number learning, on this view, would broadly benefit the performance of the ANS (i.e. increasing numerical acuity).

The possibility that the acquisition of number symbols may change ANS representations has been supported by imaging work in adults [39] and children [72], [40]. It is also consistent with the observation that adults from cultures that do not use large exact number symbols (e.g., Munduruku and Piraha tribes of Amazonia) have somewhat less precise ANS representations than adults in numerate cultures [70], [38]. Furthermore, preliminary data from a population who experience delays in language learning—deaf children born to hearing parents—reveals impoverished ANS acuity relative to their age-mates as well as delays in verbal number acquisition [73]. Collectively, these findings suggest that verbal number might indeed be a driver of subsequent development in refining or accessing ANS representations.

While the current study cannot, on its own, definitively rule out any of these relationships, it takes a first step toward understanding this relationship by characterizing, with much more specificity than previously available, the locus of the link between *changes in ANS acuity* and *changes in verbal number knowledge*. Certain hypotheses are more plausible in light of the current data. For example, Hypotheses 1 and 3, which both posit specific relationships between ANS representations and number words, are less plausible than Hypotheses 2 and 4, which both emphasize general improvements in the function of the ANS. As noted above, the dot-comparison task used quantities outside of the counting range of even CP-knowers, so children’s performance on the ANS task could not have relied on such mappings. Furthermore, we found no relationship between the quality of word-number mappings on Fast Cards and the ANS acuity measures. Therefore, it is not clear how explanations based on specific word-ANS mappings can account for the data.

Although longitudinal designs hold the promise of uncovering temporal order effects of changes in number word knowledge and ANS acuity, this study cannot definitively address the direction of causal influence between verbal and non-verbal number representations (i.e., decide between Hypothesis 2, ANS acuity first, and Hypothesis 4, cardinality first). The data do, however, show that the largest changes occurred at or after the session where a jump from subset-knower to CP-knower was observed. The current data also suggest that determining an answer to the temporal order effects will require dense sampling of children’s abilities surrounding the time just before and just after they come to understand the cardinal principle (e.g., daily testing). A microgenetic study using in-home testing with parent supervision, or a training study, would be valuable approaches here.

It also remains possible that maturational change in ANS acuity could support the acquisition of number language. For example, children’s confidence in their own ANS representations [74] could play a role in inspiring the abductive inferences required in mastering the cardinal principle. One way this could work is if a sense of increasing numerical magnitudes within the ANS inspired a number word learner to build an extension of their number word mappings to higher numerosities. Furthermore, emerging evidence shows that ANS acuity in infancy predicts mathematical knowledge in preschool [18], and that ANS-related training boosts performance on symbolic math tasks [65], [64]. Such results support the idea that changes in the ANS might potentially play a causal role in the development of symbolic mathematical abilities.

## Conclusion

The representation of large exact quantities is a remarkable achievement in children and foundational for further mathematics learning. The issue of how and when number word meanings are integrated with approximate number representations in the ANS is important for understanding how humans first come to represent large exact cardinal values [75], [76], [27], [14], [77], [41]. Recent research on this topic indicates that the causal arrows likely run in both directions: the acquisition of symbolic math is both predicted by individual differences in ANS acuity in infancy [18] and the learning of symbolic math may cause further changes in approximate number representations [38], creating a thorny puzzle for researchers and requiring a detailed look at how these systems come to be integrated. Here we address one piece of this complex story, by showing that individual children’s acquisition of the cardinal principle is tightly linked in time to a marked improvement in ANS acuity.

## Supporting Information

S1 DataSet Full Data Set With Scores on ANS and Verbal Number Knowledge Measures. (XLSX)
